# Data Efficient Reinforcement Learning for Integrated Lateral Planning and Control in Automated Parking System

**DOI:** 10.3390/s20247297

**Published:** 2020-12-18

**Authors:** Shaoyu Song, Hui Chen, Hongwei Sun, Meicen Liu

**Affiliations:** School of Automotive Studies, Tongji University, Shanghai 201804, China; s.song@tongji.edu.cn (S.S.); 1931574@tongji.edu.cn (H.S.); meicenliu@tongji.edu.cn (M.L.)

**Keywords:** automated parking system, model-based reinforcement learning, data efficiency, truncated Monte Carlo tree search, artificial neural network

## Abstract

Reinforcement learning (RL) is a promising direction in automated parking systems (APSs), as integrating planning and tracking control using RL can potentially maximize the overall performance. However, commonly used model-free RL requires many interactions to achieve acceptable performance, and model-based RL in APS cannot continuously learn. In this paper, a data-efficient RL method is constructed to learn from data by use of a model-based method. The proposed method uses a truncated Monte Carlo tree search to evaluate parking states and select moves. Two artificial neural networks are trained to provide the search probability of each tree branch and the final reward for each state using self-trained data. The data efficiency is enhanced by weighting exploration with parking trajectory returns, an adaptive exploration scheme, and experience augmentation with imaginary rollouts. Without human demonstrations, a novel training pipeline is also used to train the initial action guidance network and the state value network. Compared with path planning and path-following methods, the proposed integrated method can flexibly co-ordinate the longitudinal and lateral motion to park a smaller parking space in one maneuver. Its adaptability to changes in the vehicle model is verified by joint Carsim and MATLAB simulation, demonstrating that the algorithm converges within a few iterations. Finally, experiments using a real vehicle platform are used to further verify the effectiveness of the proposed method. Compared with obtaining rewards using simulation, the proposed method achieves a better final parking attitude and success rate.

## 1. Introduction

Automated parking systems (APSs) are important due to their great potential to reduce accidents in narrow urban parking spaces and increase parking space use [1,2]. For all APS platforms, the intelligent vehicle must generate its motion after the parking space is detected by the on-board sensors system, such as Around View Monitoring (AVM) [3,4] and Light Detection and Ranging (LiDAR) [5]. The conventional motion generation method for APS is the path-velocity decomposition method [6], where the parking task is decomposed into a kinematic sub-problem and a dynamic sub-problem, which are solved by path planning and path-following methods, respectively. However, these systems are usually not flexible in dealing with real-time perceptual information. The motion generation of APS was solved indirectly in the two stages, which was not human-like and sometimes troublesome. The deviations between the planned path and real path may cause a collision between obstacles and the vehicle. Meanwhile, the algorithms are usually rule-based and do not use the historical parking data to improve their own abilities.

To solve these problems, data-driven reinforcement learning (RL)-based APSs have been developed [7,8,9,10]. RL includes a model-based method and model-free method [11]. Model-free RL has achieved acceptable control performance for APS [8], in which the algorithm learns to steer by directly trying actions in an attempt to attain a maximum accumulative reward. Unfortunately, this method requires thousands of real-time interactions for applications. Data inefficiency makes model-free RL impractical and limits its application in parking scenarios, in which the vehicle is required to quickly acquire driving skills. Model-based reinforcement learning [10] was used to realize multi-objective optimization and get rid of human experience. However, as the action was determined by the results of simulations, this method heavily relied on the accuracy of the vehicle model. A large number of trials were necessary for the obtaining and verification of the vehicle model. Furthermore, it cannot continuously learn using the limited number of parking data collected on the controlled object with the unknown model to further improve the ability. Data efficiency refers to the number of needed data to get a stable performance. Little research has been conducted to address the data efficiency for RL-based APS especially for model-based RL APS or other automatic driving scenes while maintaining the continuous learning ability. Regarding model-based RL technology, AlphaGo [12] utilized the basic rules in the game of Go and function approximation to get the state value function and to defeat the human player by self-play, which inspired the combining of [10] and the state value function to overcome the shortcoming of model-based RL parking system.

In this paper, we propose a data-efficient reinforcement learning (DERL) algorithm to solve APS lateral planning and control in an integrated method. Continuous learning is realized by fitting a state value function. The approximate modified policy iteration (AMPI) [13] was used to take advantage of the state transformation function to construct the state value function using a priori knowledge of the vehicle kinematics, thus showing high data efficiency. The AMPI method used in this paper is the Monte Carlo tree search (MCTS) [14,15], which has also been used in AlphaGo [12] and AlphaGo Zero [16]. In a Monte Carlo parking setup, the reward is given at the end of the trial. Designing a reward function for the final performance is simpler and more objective than offering immediate rewards. Unlike the model-based method used in [10], the requirement for the accuracy of the vehicle model can be reduced by limiting the use of simulation in the search process. MCTS uses the estimation of the state value and the probability distribution of candidate actions. In addition to the one artificial neural network (ANN) used to learn the probability distribution of the action with high reward [10], another value ANN is used to predict the state value. To further improve the data efficiency, several methods are proposed. The update direction of the ANN can be enhanced by adding a weighting coefficient with the return of the parking trajectory. To better use the ANN, an adaptive exploration scheme is also developed to encourage exploration in the direction recommended by the ANN. The vehicle model is used to generate imagination rollouts, which is supplemented with real parking data. The reasons for using DERL are threefold: First, the Monte Carlo structure is suitable for parking tasks [10], where rewards are sparse. Second, the use of the value ANN can truncate the search process and avoid the accumulation of errors. Third, a vehicle model can be used to speed up the learning, which finds a local optimal solution in a rolling-horizon manner. Compared with the path planning method using a continuous curvature curve, the proposed method can successfully park a vehicle in a smaller parking slot in one maneuver. The principal contributions of the proposed method are fourfold:

- A model-based RL addressing the ability to continuous learning for APS is proposed. A truncated MCTS algorithm is used to implement this method, of which the data efficiency is improved by designing an adaptive exploration encouragement factor and weighted policy learning to enhance the network updating toward the direction of the trajectory with high return. Thus, the influence of vehicle model and sensor systems can be reduced.
- The proposed approach continuously plans and controls motion, which has lower requirements for the perception system at the initial parking position, in principle. For its data-driven feature, the parking trajectory given by the pose estimator can be used to improve the system’s performance.
- The novel training method consists of two phases of learning for the greedy parking lateral action policy—classification network and state value estimate—fitting network, which do not need human input at the early stage of APS training.
- The proposed method is verified on a full-size vehicle and is shown to achieve acceptable performance without time-consuming training on the real platform.

The remainder of this work is organized as follows: Section 2 introduces the related works. Section 3 introduces the problem definition on the basis of the system structure. The defined optimal control problem is solved in Section 4. The results and discussion of simulations and real-vehicle experiments are provided in Section 5 and Section 6, respectively. Finally, Section 7 draws the conclusions.

## 2. Related Work

### 2.1. Environmental Perception

A parking area is defined as the empty space formed by two obstacles or painted lines. For an obstacle-formed parking space, the range sensor-based methods were developed [5,17]. Ultrasonic sensors and LiDAR can be used to fit the shapes of obstacles. Although the detection accuracy has been improved by a recent work [5], these methods could not track the parking space after the parking motion started. The vision-based approaches were developed for the line-formed parking space. The AVM system can continuously track the parking slot. To obtain the slot marking result, deep convolutional network-based segmentation methods were proposed [1,4]. For the tracking of the object, the prediction accuracy is deteriorated by noises, and the occlusion should be considered. A kernelized correlation filter was used to explore intrinsic features from background-interfered samples [18]. During the parking, simultaneous localization and mapping (SLAM) was developed to localize the vehicle at the centimeter level [3].

### 2.2. Motion Generation

Many path planning and path-following control methods have been proposed to generate parking motion. The former includes numerical optimizations, A* search, rapidly exploring random tree (RRT) family, and curve planners. In the optimization methods, local optimization [1,19] was developed to find the short-range path. Although the global optimality cannot be guaranteed, they usually generated feasible paths in a short time. In contrast, the global optimality method [20] took a long run time to find the optimal parking maneuver. The A* search method [21] discretized the search space, which guaranteed the completeness and optimality. However, the selection of the grid size requires expert knowledge. RRT methods [22] can plan in the continuous space using heuristic information. Curve planners [23] generated continuous curves by combining lines and arcs. For path following methods, linear quadratic regulator (LQR) [24], sliding mode control [25], and fuzzy controller [26] were used. These control methods, however, cannot deal with system constrains, such as the actuator limits of the steering system. To ensure that the designed path can be roughly followed, path planning must construct an admissible path bounded by the maximum curvature and curvature change rate under the conservative speed hypothesis. Although the curvature may be continuous, the actual trajectories still deviate from the expected path owing to the tracking errors. The system is not flexible enough to deal with real-time perceptual information and maximize the vehicle’s adaptability.

To improve the adaptability of APS, model-free reinforcement learning (RL)-based APS have been developed, in which the algorithm learns to steer by directly trying actions in an attempt to attain a maximum accumulative reward. The advantage of RL lies in its ability to cope with system constraints, as well as its data-driven and human-like continuous learning manner. The RL method explicitly takes system constraints, typically in the form of actuator limits, into consideration at each time interval. The algorithm makes decisions based on the real-time motion states and the perceptual information. Meanwhile, the errors of path-following control can be fundamentally avoided by integrating planning and control using RL, which increases the upper bound of the system. Deep Q-learning (DQN) [27] has been used to learn the heuristic information for Hybrid A*, which provides computational advantages over conventional path planning. Different setups for DQN-based motion planning [7] have been compared, which demonstrated its acceptable runtime performance on several devices. The deep determinist policy gradient (DDPG) [9] with preview control, which relies on a reference signal, has been proposed to solve the optimal control problem of vertical parking. DDPG [8] with manual guide exploration and different control cycle re-training pipelines has been used to achieve reactive end-to-end parking in a real vehicle platform. However, model-free RL-based APS requires large amounts of data, which makes the training on the real vehicle infeasible and unscalable.

**Figure 1:**
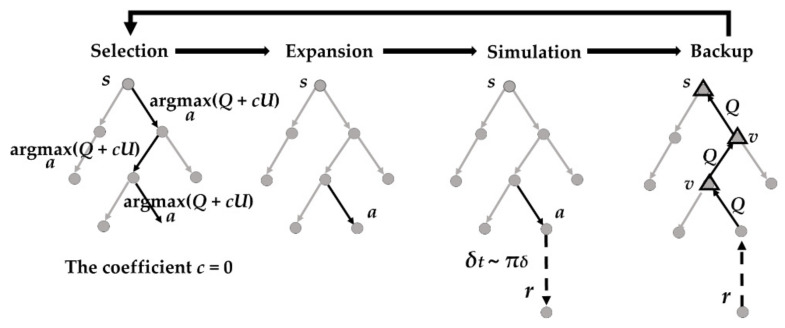
The tree search used in the previous work [10].

In the model-based RL method, MCTS with memory chain was proposed in [10] and [28] to get rid of the human experience. As shown in Figure 1, the MCTS includes selection, expansion, simulation, and backup steps. The state value was obtained by multi-step simulation from the leaf node. The probability to choose action in the simulation was given by a policy ANN, of which the inputs were the parking states, the output was the probability distribution. During the action selection in the tree search, the weight of policy was set to 0 (i.e., the coefficient *c* in Figure 1). The system identification was used to improve the model accuracy. The multi-objective optimality was realized by combining MCTS and longitudinal and lateral policies. A large amount of parking data can be obtained. However, this method has high requirements for the accuracy of the vehicle model, which limits its application.

## 3. System Structure and Problem Definition

### 3.1. Structure of Automatic Parking System

**Figure 2:**
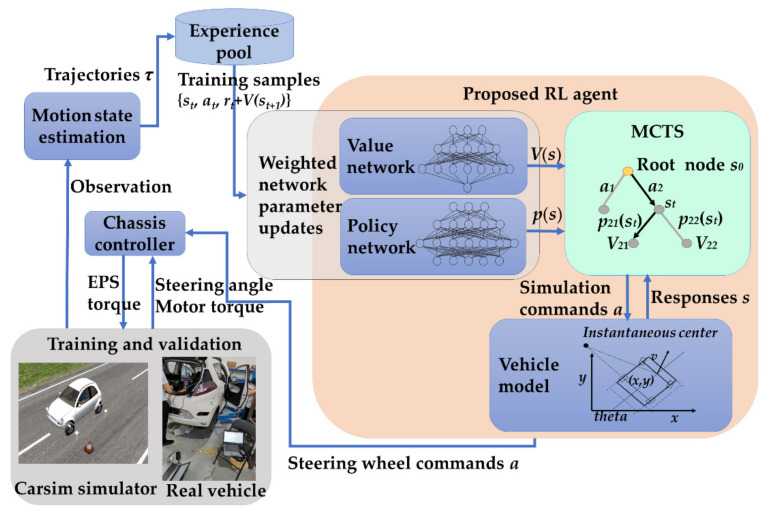
Overview of the reinforcement learning (RL)-based parking architecture in which Monte Carlo tree search (MCTS) was used as the agent. The steering action was realized by the electric power steering (EPS) system. The associated speed command for each steering action is the function of distance, and designed to stop within the safety threshold [10].

The overall architecture of the method is shown in Figure 2. The input of the proposed RL agent is the motion state and the output is the steering wheel angle command. At each control interval, the control variable is calculated based on the real-time state. Together with the steering wheel angle command, the torque request is obtained by the controller of the vehicle steering system. Carsim and a real vehicle are used to validate the learning algorithm. Kalman filtering is used to process motion sensor data.

### 3.2. Problem Definition of Parking

Markov decision processes (MDPs) consist of a tuple <*S*, *A*, *P*, *r*, *γ*>, where *S* is the state, *A* is the action, *P* is the transition probability distribution, *r* is the reward function, and *γ* ∈ (0, 1] is the discount factor. The goal is to optimize over a return on *K* steps:

(1) $$R(\tau )=\sum_{k=t}^{t+K}\;\gamma^{k}\;r(s_{k},\;a_{k}),$$

where ***s****_k_* and *a_k_* are the state and action at time *k*, respectively, and *τ* = (***s****_t_*, *a_t_*, *r_t_*_*+*__1_, ***s****_t_*_*+*__1_, *a_t_*_*+*__1_, *r_t_*_*+*__2_, …) is a complete trajectory. Let ***π*** denote a policy to choose an action by the agent. The state value function, *V**_π_***(***s***), is the expectation of return on ***s***:

*V**_π_***(***s***) = E***_π_*** [*R*(*τ*)|*S*_*t*_ = ***s***]. (2)

The goal of RL is to obtain the optimal policy π*, such that:

*V*_π*_(***s***) = max***_π_****V****_π_***(***s***). (3)

Similarly, the value function for an action *a* and state ***s*** is defined as a state-action value function:

*Q****_π_***(***s***, *a*) = E***_π_***[*R*(*τ*)|*S*_*t*_ = ***s***, *A*_*t*_ = *a*]. (4)

The optimal *Q*_π__*_(***s***, *a*) can be written as:

*Q*_π*_(***s***, *a*) = E[*r*_*t*_ + *γV*_π*_(***s***_*t*_ + _1_)|*S*_*t*_ = ***s***, *A*_*t*_ = *a*]. (5)

The main criterion for parking is the final position of the car. The desired end position for parallel parking is parallel to the connecting line of the front and rear obstacle vehicles, while the parking depth should be ensured such that the wheel avoids hitting the edge of the parking space. As the parking depth *y* and orientation *θ* approach target values, the reward should increase. The gradient near the target should be high, in order to place a focus on these states. Accordingly, the reward function is:

*r*(*k*) = *R_y_* + *R_θ_* + *R_a_* + *R_safe_*, (6)

with:

(7) $$R_{y}\;=\;\frac{-20000}{1+e^{-c_{1}\;\times \;|\;y\;-\;yt\;|}}\;+\;20000,\;R_{\theta }\;=\;\frac{-20000}{1+e^{-c_{2}\;\times \;|\;\theta \;-\;\theta t\;|}}+\;20000,\;R_{a}\;=\;c_{3}\;\times \;\sum_{i=0}^{k}\;|a_{i}-\;a_{i-i}|,\;\;R_{\mathrm{safe}}\;=\;-10000,\;$$

where the reward components *Ry R_θ_* of the parking depth and orientation are sigmoid-function-like S curve, which has a bounded function value. The term *R_a_* is used to encourage finishing the task with relatively small steering action. The constants *c*_1_, *c*_2_, and *c*_3_ are scaling factors. The first two coefficients determine the decreases in the speed of reward regarding parking depth error and angle error, respectively. The *c*_3_ determines the weight of steering wheel actions. If a collision occurs, a − 10,000 punishment is given.

The front-wheel steering single-track kinematic vehicle model is extensively used for low-speed parking (i.e., when the lateral acceleration is below 0.4× *g*). Sideslip can be ignored. The state transition function [6] for the agent’s internal simulation is:

*x′* = *v*(*t*)cos*θ*(*t*) *y′* = *v*(*t*)sin*θ*(*t*) *θ′* = *v*(*t*)tan*φ*(*t*)/*l*. (8)

The front wheels are simplified to one steering angle. Assuming there is no sliding, given a steering angle, Ackermann steering geometry ensures that the vehicle will travel in a circle centered at the intersection of the rear central axis and front wheel perpendicular lines.

## 4. Data-Efficient RL Algorithm Design

Next, the data-efficient learning approach and its algorithm for parking planning and control are introduced. The parking accuracy issue was solved by introducing the DDPG algorithm [29] for end-to-end automatic parking [8]; however, obtaining large amounts of data is expensive. To increase data efficiency, a model-based truncated MCTS for approximate modified policy iteration is used, followed by three improvement schemes. 

### 4.1. Truncated MCTS for Approximate Modified Policy Iteration

#### 4.1.1. Approximate Modified Policy Iteration

The idea is to use the function space approximating the state value function of a policy (policy evaluation, E-step), followed by finding better policies using greedy steps based on the recent evaluation (policy improvement, I-step). The evaluation-improvement pair [13] is as follows:

*V****_πk_***(***s***) = E***_π_***_*k*_[*r*_*t* +__1_ + *γV′_**π**k_>* (*S*_*t* +__1_)|*S*_*t*_ = ***s***], E-step, (9)

*π_k+_*_1_(***s***) = arg max*a Q****_π_****_k_*(***s***, *a*) = arg max*a* E [*r_t_ + γV****_π_****_k_* (*S_t +_*_1_)*|S_t_ = **s***, *A_t_ = a*], I-step. (10)

AMPI starts from an initial value *V*_0_ and initial policy ***π***_1_. At iteration *k*, the new state value function *V_k_* is built using the Bellman operator on *V_k_*_− 1_. Then, the state value function is used to obtain the action value. A new policy is obtained by choosing a stronger action *a*. If the policy improvement theorem *Q**_π_**_k_*(***s***, *π_k_*(***s***)) ≥ *V**_π_***_(*k*__− 1)_ is satisfied, the new policy ***π****_k_* converges to the optimal policy. For each policy pair and estimate of the state value function, a set of rollouts is generated to build *N* training samples {***s****_t_*, *a_t_*, *r_t_* + *V*(***s****_t_* *+*_1_)}. Non-linear approximators *P*′ and *V*′ can be used to represent the greedy policy and state value function, respectively. The policy *P*′ to be trained is expressed as a classifier, while *V*′ is a regressor. The goal of Equation (9) is to minimize the empirical errors:

(11) $$L_{V^{\prime }}(s)\;=\;\arg\;\min_{\;V^{\prime }}\;[\frac{1}{N}\sum_{i\;=\;1}^{N}(V^{\prime }(s_{i})\;-\;(r_{i}\;+\;V(s_{i\;+\;1})){)}^{2}],$$

(12) $$L_{P^{\prime }}(\pi^{\prime })\;=\;\arg\;\min_{P^{\prime }}\;[\frac{1}{N}\sum_{i\;=\;1}^{N}(P^{\prime }(s_{i}|\pi^{\prime })\;-\;a_{i}{)}^{2}].$$

The algorithm iterates between policy evaluation and policy improvement. Algorithm 1 provides a complete description of the approximate modified policy iteration.
**Algorithm 1** Approximate Modified Policy IterationInput: Value function *V_0_*, policy function *P_0_*Output: Optimal value function V*, optimal policy function P*1. **for**: *l* = 0, 1, 2, …, *L*
**do**2.    Generate rollouts using *V_l_* and *P_l_* by Equation (10)3.    Approximate value by Equation (11)4.    Approximate policy by Equation (12)5. **end for**

#### 4.1.2. Truncated MCTS Guided by Artificial Neural Networks

In this study, policy improvement and policy evaluation were implemented using MCTS. The parking trajectories generated by reinforcement learning are used to train a value network and a policy network (i.e., the policy evaluation step). Then, the tree search is used to select an action stronger than the mean value of the output given by the networks (i.e., the policy improvement step).

- Truncated MCTS

**Figure 3:**
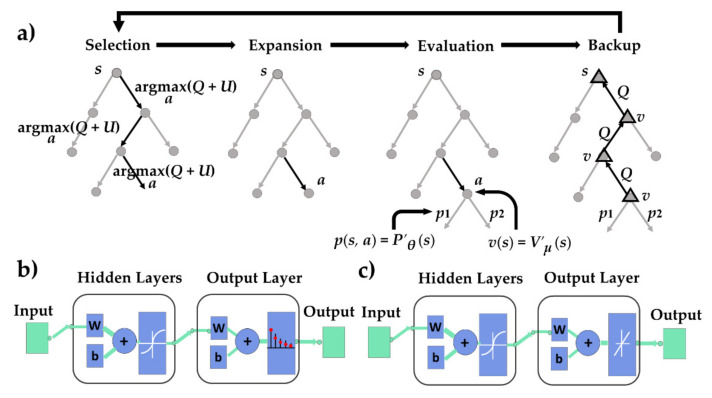
The truncated MCTS guided by the policy and value networks: (**a**) Truncated MCTS; (**b**) structure of the policy network *P*’*_θ_* (***s***); and (**c**) structure of the value network *V*’*_μ’_* (***s***).

When the agent encounters a new state, forward sampling sequences ***s***, *a*, and *r* are used to estimate the value functions. In MCTS, the state ***s*** of the agent is viewed as a node, while the edge (***s***, *a*) stores statistical information of the state-action pair. The search tree is incrementally built from the root node to the target state by adding promising nodes to the tree based on simulated trajectories. The *Q*-value is computed as:

(13) $$Q(s,a)\;=\;\frac{1}{1+N(s,a)}\sum_{i\;=\;1}^{N}\;\bar{I}(s,\;a)z_{i},$$

where *N*(***s***, *a*) is the visit number of action *a* from state ***s***, Ῑ (***s****, a*) is the bool variable indicating whether *a* is selected, and *z_i_* is the reward of the *i* th sampling. In Figure 3, a truncated variant of the MCTS is used [14]. Compared with the frame of the previous works [10], this study uses the value function approximation to truncate the simulation in MCTS such that the model errors cannot be accumulated. Besides the policy ANN, the value ANN (i.e., the ANN in Figure 3c) is also used to select action in DERL. The statistical value is stored in the edge:

{*P*(***s***, *a*), *N*(***s***, *a*), *W*(***s***, *a*), *Q*(***s***, *a*), *UCT*}, (14)

where *UCT* is the value of the upper confidence bound for trees. Each sampling selects an edge from the root node, according to *UCT*:

(15) $$a_{t}(s)\;=\;\arg\;\max\;a\;(Q(s,\;a)\;+\;{c_{puct}}_{\;}P{\left(s,\;a\right)}^{\mu }\;(\frac{\sum_{b}\;N(s\;,b)}{1+N(s\;,a)}{)}^{0.5}),$$

where the second term (denoted as *U*) is for controlling exploration and *μ* is an adaptive coefficient to bias the search. The smaller the value of *μ* is, the stronger the effect of *P*(***s***, *a*). When MCTS traverses to a leaf node, child nodes are added to the tree and the new nodes are evaluated by the value network *v*(***s***) = *V′_μ′_*(***s***). The total visit number *N*(***s***, *a*) and the total value of the action chain from the root node are updated as *N*(***s***, *a*) = *N*(***s***, *a*) + 1, *W*(***s***, *a*) = *W*(***s***, *a*) + *v*(***s***). When the computation budget is reached, the real action is selected by [12]:

(16) $$\pi (a|s)\;=\;\frac{N{\left(s,\;a\right)}^{1/\tau }}{\sum_{b}\;N{\left(s,\;b\right)}^{1/\tau }},$$

where *τ* is a temperature coefficient and *b* is the root node’s child node.

- ANN Approximation for State Value and Greedy Policy

Two completely connected forward networks were built for policy evaluation. The state value network represents the value of the current vehicle states, regarding the final overall performance. The policy network represents the instantaneous reactive experience learned from the training iteration and provides information to the MCTS for balancing exploration and exploitation. The inputs and outputs of the ANNs are listed in Table 1. The inputs of *V*′*_μ′_* (***s***) include the vehicle pose, steering wheel angle, and node’s brother number ***s*** = [*x*, *y*, *φ, ψ, br*], where the output is the state value estimation *Vest*. The training is implemented in MATLAB, where the set of parameters for the ANNs is selected empirically for different models.

The target state value, *V*, is crucial for estimating the true action-state value *Q*, in order to determine the priority of adding nodes to the tree, and to select the action in the tree for actual execution. The value of *V* is obtained by Equation (6). The optimality and convergence of the policy iteration rely upon the fitting accuracy of value network *V*′*_μ_* (***s***) with parameters *μ′*. The network was trained using the Levenberg–Marquardt method by minimizing:

(17) $$L(V_{est},\;V)=\;\frac{1}{N}\sum_{i=1}^{N}=\;{\left\|Vest\;-\;V\right\|}_{2}^{2}.$$

The policy network *P*′*_θ_* (***s***) was used to directly recommend the action probability distribution, such that the brother number was not necessary for the current state. For multiclass classification problems, the input is ***s*** = [*x*, *y*, *φ, ψ*] and the output ***p***(***s***, ***a***) is the probability for each action. Cross-entropy loss is used:

(18) $$H(p,\;q)=-\sum_{i=1}^{N}\;p(x_{i})\log(q(x_{i})),$$

where *q*(*x_i_*) is the target one-hot vector of action category.

### 4.2. Data-Efficient Promotion Methods for RL

To promote data efficiency during the training process, three ideas were proposed. First, we encouraged the networks to update towards the high-return direction though weighting policy learning with trajectory returns [30]. Secondly, the simulation-based search experience was merged with a real episode to extract parking information. Third, a novel training pipeline customized for MCTS was proposed.

#### 4.2.1. Policy Learning by Weighting Exploration with Trajectory Returns 

For state ***s***, if the action value of the new policy is higher than the old policy’s state value *Q**_π_**_k_*(***s***, *π_k_*_+ 1_(***s***)) ≥ *V**_π_**_k_*, the algorithm is convergent [11]:

*V**_π_***(***s****_t_*) ≤ *Q**_π_***(***s****_t_*, π′(***s****_t_*)) = E[*r_t_* + _1_ + *V**_π_*** (***s****_t_* + _1_)|*A_t_* = π′(***s****_t_*)] = E***_π′_*** [*r_t_* + _1_ + *γV**_π_***(***s****_t_* + _1_)] ≤ E***_π′_*** [*r_t_* + _1_ + *γQ**_π_***(***s****_t_* + _1_, ***π′***(***s****_t_* + _1_))] ≤ E***_π′_*** [*r_t_* + _1_ + *γr_t_* + _2_ + *γ*^2^*r_t_* + _3_ + *γ*^3^*r_t_* + _4_ + …] = *V****_π′_***(***s****_t_*). (19)

*V**_π_*** can be updated to maximize its calculated value. Consider a trajectory *τ*_1_*, τ*_2_*, τ*_3_*, …, τ_K_* for each iteration. The total expected return is:

(20) $$J(\pi )\;=\;\int_{k=0}^{K-1}p_{\pi }(\tau_{k})R(\tau )d\tau ,$$

where *p****_π_***(*τ_k_*) is the probability distribution of trajectory *τ_k_* under policy ***π***. For the original policy iteration, all the trajectories are used to evaluate the policy. In the field of model-free RL, the policy gradient method [8,9] takes the partial derivatives of the expected return of the network parameter such that the return is maximized. Inspired by this, if a new distribution *p′*(*τ_k_*) considering the trajectory return is applied to *τ*, the new total expected return might be higher [30]:

(21) $$\int_{k=0}^{K-1}{p^{\prime }}_{\pi }(\tau_{k})R(\tau )d\tau \;>\;\int_{k=0}^{K-1}p\pi (\tau_{k})R(\tau )d\tau .$$

As discussed in Kober et al. [30], this distribution can improve the lower bound in policy learning. The rule to update the network associated with the weighting factor is:

(22) $$\theta_{l+1}\;=\;\theta_{l}\;+\;\alpha^{\prime }\frac{R(\tau k)}{\sum_{k=1}^{K}R(\tau k)}\partial_{\theta l}J(\theta_{l}).$$

Differing from the original on-policy AMPI where the evaluated policy is used to make decisions, our implemented method is off-policy, as it records the entire trajectory. The *K*_s_ trials of the highest historical returns are used to improve the current policy.

#### 4.2.2. Experience Augmentation with Imagination Rollouts

Apart from direct reinforcement learning from real experience, when the environment model is available, simulated experience can also be used to train the RL. The model of a vehicle contains a priori information of the environment with which the agent/algorithm is interacting. Extracted information from the vehicle model benefits the construction of the value function, which is important for organizing the MCTS. Therefore, imagination rollouts are mixed with the real trajectory to train the network *V*′*μ*(***s***).

**Figure 4:**
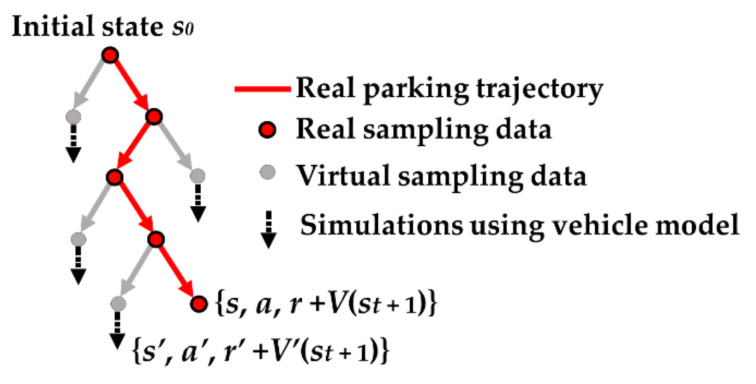
Parking data imagination.

The vehicle model is available in vehicle engineering. As the parking task involves driving the car at low speed, the single-track kinematic model has been extensively used in APS for small lateral acceleration [31]. The data augmentation scheme is shown in Figure 4. After the real trajectory (red line in Figure 4) is generated, the brother nodes of the parking trajectory are used as virtual root nodes. MCTS performs the simulation using the vehicle model (Equation (8)) on these branch points until the parking process terminates. The final reward, *r_T_*, is discounted and accumulated to obtain the unbiased estimate of the return (Equation (2)) on the virtual root nodes. The data of the training samples {***s****_t_*, *a_t_*, *r_t_* + *V(**s**_t_* *+*_1_*)*} can be increased in this way.

A small action difference makes it hard to produce a significant value distribution difference, as the vehicle speed and control interval are small during parking. Therefore, each move from the root node is repeatedly sampled many times; we found that 10 times was enough. The simulation number, which was used to obtain the unbiased estimate of the state value, was chosen by considering the number of computer kernels. In our implementation, this number was 36.

#### 4.2.3. Warm Start with Pre-Trained RL Model

**Figure 5:**
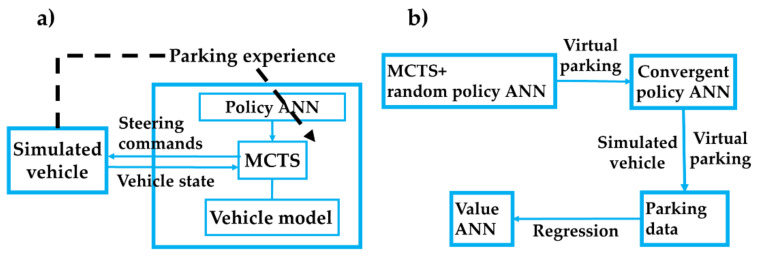
Training pipeline of the proposed method with (**a**) the pre-train method using MCTS and the policy ANN [10]; and (**b**) the training pipeline to obtain the value ANN.

The proposed method consists of three parts: the value network, the policy network, and the MCTS. As the convergence of RL is difficult, a novel training pipeline in Figure 5 was proposed to warm-start the overall method before online training using the value network and MCTS. Figure 5a is used to obtain the convergent policy network in Figure 5b. Step by step, tuning the parameters is easier.

Differing from Figure 3, where the value of the leaf node was obtained by *V*′*_μ_*(***s***), the MCTS in the pre-train model used the policy network *P*′*_θ_*(***s***) for simulation, the detailed process to obtain the simulation results can be found in Zhang et al. [10]. The kinematic vehicle model was used for simulation. First, the selected parking experience was used to train a policy network. Then, MCTS controlled the vehicle model to park under the guidance of the policy network. The simulation data were collected to train the value network in Figure 5b. The data can also be obtained by other methods, such as the optimization-based method. The reward was determined by the reward function and the simulated terminal state.

The overall algorithm is shown in Algorithm 2. The coefficient *μ* in Equation (15) was obtained by experimental simulation. We determined the direction of the recommended policy action at the root node. If the current node coincided with the recommendation, *μ* was set as 0.5, in order to assign a higher weight to *P*. In the parking application, this recommended action was abstracted as {*increase_ angle, decrease_ angle, no_action*}.
**Algorithm 2** Proposed overall algorithmInput: Value function *V_0_*, policy function *P_0_*Output: Optimal value function V_*_, optimal policy function P*
1. **for**: *t* = 0, 1, 2, …, *T*
**do**2.  Generate rollouts using MCTS guided by *P_t_*, Equation (15), where *Q* is obtained by simulation3.  Approximate policy *P_t +_*_1_ by Equation (12) using the loss function Equation (18)4. **end for**5. Generate rollout samples *τ* = {*s_t_*, *a_t_*, *r_t_* + *V*(*s_t +_*_1_)} using MCTS and *P_T +_*_1_6. Approximate value network *V*_0_ by Equation (11) using the loss function Equation (17)7. **for**: *i* = 0, 1, 2, …, *I*
**do**8.   Generate rollouts using MCTS guided by *P_i +_*_(*T +* 1)_ and *V_i_*_+ 0_, Equation (15)9.     **for**: ***s*** = ***s*_0_**, …, ***s***_t_
**do**10.        calculate recommend action by *P_i +_*_(*T +* 1)_11.        pr **=** {increase_ angle, decrease_ angle, no_action}12.        execute MCTS obtain *a*13.        **if**
*termination*14.          *r* = *rewardfunction*(***s***_t_)15.        **end if**16.     **end for**17.  Experience augmentation as Figure 418.  Approximate value by Equations (11) and (17)19.  Approximate policy by Equations (12) and (18)20. **end for**21. **function** MCTS(***s***_0_, *P*, *V*, *pr*, *char*)22.  execute Equations (15) and (16) with23.     **if**
*char* = = ’*pretrain*’24.        *v*(***s***) = simulation from ***s*** to end with *P*25.     **else**26.        *v*(***s***) = *V*(***s***)27.     **end if**28.  **if**
*a_child* ∈ *char*29.        *μ* = 0.530.  **else**31.        *μ* = 1.532.  **end if**33. **return**
*a*34. **end function**

## 5. Simulations 

**Figure 6:**
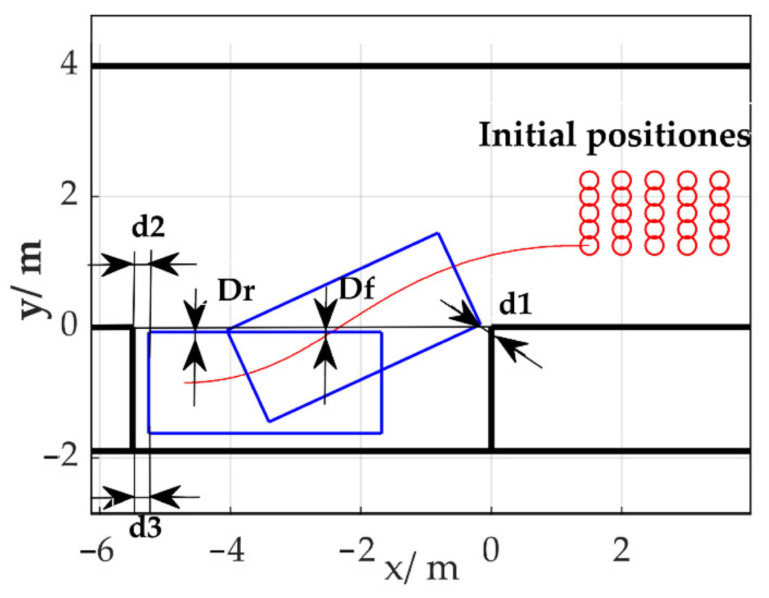
Simulation and experimental environment setup in the parking co-ordinate system (Dr and Df are the distances from rear and front tyres to the parking space edge; d2 and d3 are the distances from left and right corners to the rear obstacle; d1 is the minimum distance to the front obstacle).

For convenience of comparison, the initial poses for parking were fixed. They were evenly distributed in *x* = [1.5 m, 3.5 m], *y* = [1.25 m, 2.25 m] with 25 positions. The initial heading angles were set to zero. Parallel parking was considered in this study, in order to determine the effectiveness of the proposed learning method. The simulation conditions were as follows: the length of the parking space was 5.5 m with a target parking pose of −0.85 m and 0° in *y* and *θ*, respectively, in the parking space co-ordinate system (as shown in Figure 6). The entire set of parameters are listed in Appendix A. 

### 5.1. Feasibility of the Learning Algorithm

We performed simulations to study the effectiveness of the proposed method. To this end, the control object was first set as a single-track kinematic model, which was used to quickly obtain the pre-train model. Then, the policy network was used to obtain the value network. The influence factor of the algorithm was investigated by controlling the Carsim vehicle. 

#### 5.1.1. Model Pre-Trained with the Policy Network and MCTS

**Figure 7:**
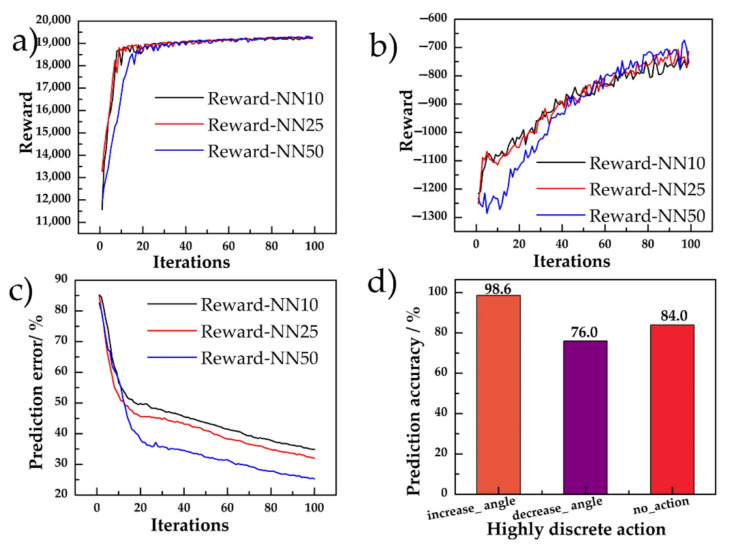
Results of the pre-train model: (**a**) Total reward using different hidden layer size of the policy networks; (**b**) reward of the steering smooth term *Ra* defined in Equation (6) for 3 sizes of policy networks; (**c**) steering wheel angle prediction error; and (**d**) fuzzy action prediction accuracy of the selected 25 × 25 hidden layer network.

The pre-trained model was used to obtain the policy network. In the reward function in Equation (6), the range of *R_y_* and *R_θ_* was 0 to 20,000, while the experimental value of the convergent −*R_a_* was 600 to 1000. The learning process for pre-training the model is shown in Figure 7. The size of the network had little influence on the final overall reward and smooth term. A 50 × 50 network was slow to converge, due to the insufficient training for the larger number of parameters. Using a relatively small network, the prediction error converged faster. During the early stage of learning, the reward increased quickly, approaching 19,000. Although the prediction error could be further reduced, the reward of the pre-train model converged in about 20 iterations, which included 500 parking simulations. Finally, the hidden layer size of the final policy network was designed to be 25 × 25, considering the real-time performance of the experiment.

The policy network’s prediction error is shown in Figure 7c,d. The final prediction error of the selected network for each exact action (resolution of 5 degrees) was over 30%. To more effectively use the learned information of the policy network, the action was blurred as {*increase_angle, decrease_angle, no_action*}. The prediction accuracy for the heuristic information of the action direction is given in Figure 7d, where the boundary for successful prediction is when the probability sum of the corresponding action output of the network is higher than 0.5. The figure indicates that the prediction accuracy after the fuzzification action was high.

#### 5.1.2. Complete Training of RL Model

**Figure 8:**
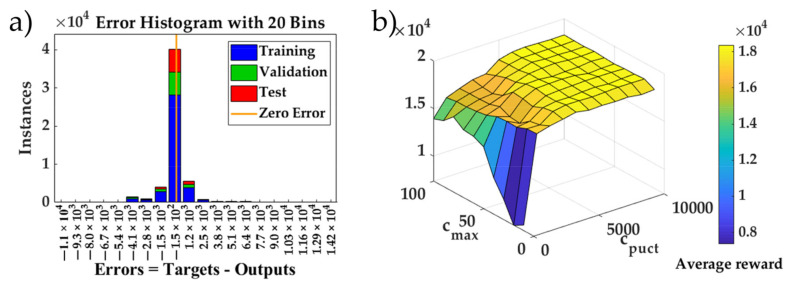
The results of the first generation of parking simulations combining MCTS with the policy network and value network: (**a**) The training error histogram distribution of the value network; and (**b**) the average reward over 25 initial parking positions for different MCTS parameters.

After the policy network was obtained, data {***s****_t_*, *a_t_*, *r_t_* + *V*(***s****_t_* *+*_1_)} were generated (Figure 4) and the rewards were used to train the value network. To reduce the influence of randomness for the training of ANN, ten networks were trained with different training processes, where the data sets were differently randomly divided by the same rate of 80% for training, 10% for validation, and 10% for testing. The ten networks were obtained using the same data of 90% in the raw data with different training/validation/testing data, 10% of the raw data was preserved to select the network. This can ensure that the 10% of the raw data is not seen by the networks during the training. The network with the lowest test error on the preserved data set was selected. The training of policy networks follows this same process. Figure 8a depicts the training process of the best value network, showing that 70% and 69.9% of the training and test sample errors were below 1000, respectively.

To study the influence of the parameters on the parking results, the performance of the proposed method over all 25 initial poses with different Monte Carlo sampling times *c_max_* and weight terms *c_puct_* in Equation (15) was evaluated using the Carsim vehicle. Figure 8b depicts the average reward of 11 different *c_puct_* values (from 0 to 10,000) of the policy network and the corresponding sampling times *c_max_* (from 10 to 100). The reward upper bound was 20,000. For *c_puct_* ≥ 6000 and *c_max_* ≥ 30, most of the reward was above 18,120; deducting the mean action punishment of 600, the residue reward was 1230 (0.82 degrees) in *R_θ_* and 50 (0.9 cm) in *R_y_*. For each *c_max_* value, the lowest reward consistently occurred when *c_puct_* = 0. This suggests that the sampling priority in Equation (15) benefitted from the guidance of the policy network. The policy network is important and complementary to the value network, in which the value prediction error was unavoidable. 

**Figure 9:**
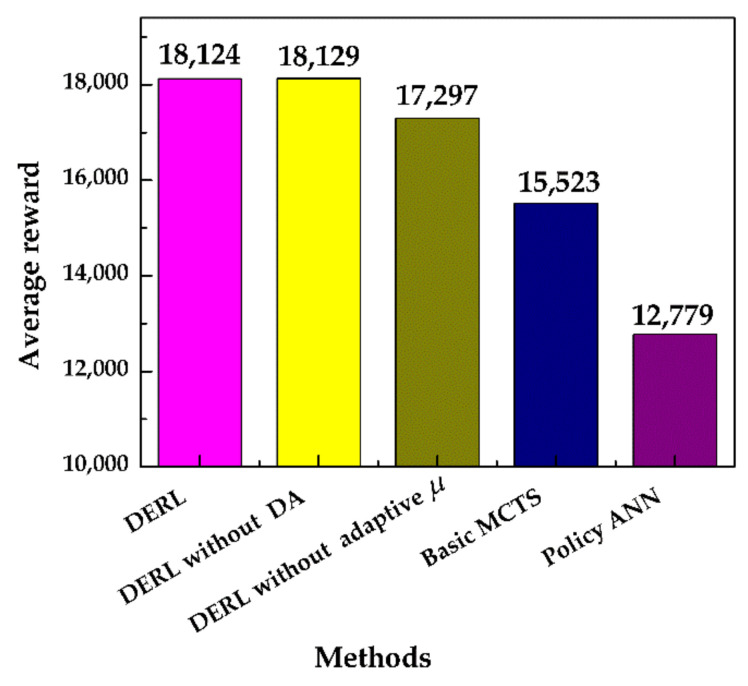
Average rewards received during training by the original algorithm and the proposed data-efficient reinforcement learning (DERL) algorithm. The basic MCTS has neither data augmentation (DA) or adaptive *μ*.

To evaluate the contributions of the proposed method to the improvement of the combined model, more simulations were conducted for comparison. We used parameters *c_puct_* = 6000 and *c_max_* = 30, as they provided a good result (see Figure 8). As shown in Figure 9, the proposed data-efficient RL achieved high average reward over 25 initial positions. By contrast, the performance of the policy network alone was the worst. This verified the contribution of MCTS and the value network. The adaptive search process *μ* of Equation (15) was the most crucial component for the DERL. Data augmentation (DA) is important, given the lack of adaptive *μ*. This suggests that increasing the weights of promising action in the same action direction as the recommended action at the root node can significantly enhance the overall performance. The reason for this is that the policy network’s prediction of the fuzzified action was accurate (see Figure 7). The proposed method benefits from the extracted information. Without this extracted information, the value network becomes more important. The reward increased from 15,523 to 17,297 with the help of data augmentation. The conventional value-based RL method is sensitive to the fitting accuracy of the value function. The result indicates that using exploration guidance can potentially conquer this shortcoming, similar to what was observed in the multi-armed bandit problem [14], where the regret of the algorithm benefited from a predictor. 

### 5.2. Comparison with Curve-Based Path Planning Method

**Figure 10:**
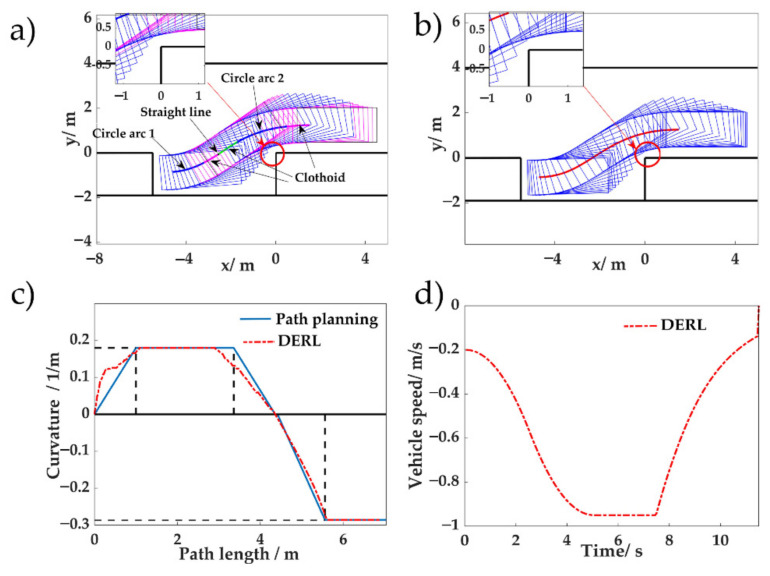
Vehicle trajectories given by different methods: (**a**) Ideal trajectory of the planned path using continuous-curvature curve-based method; (**b**) vehicle trajectory of the proposed RL method; (**c**) path curvatures of different methods; and (**d**) vehicle speed command of the proposed RL parking.

We compared curve-based path planning and the proposed method. Continuous-curvature path planning uses clothoid curves [23,24]. In the conventional path planning method for parallel parking, the vehicle is retrieved from the parking space [32]. As shown in Figure 10a**,** circle arc 1 has the smallest radius and circle arc 2 has a radius greater or equal to the minimum radius. The clothoid curves and straight lines are used as transition curves. The goal pose for continuous-curvature path planning was *y* = −0.85 m, while *θ* was determined by whether the parameter could be found by the retrieval process. The initial goal pose was located at the center of the parking space. If retrieval safety could not be satisfied, *x_goal_* was reduced and *θ_goal_* was increased. The safety buffer distance for both curve-based path planning and the proposed RL-based method was 0.25 m in the parking slot and 0.15 m at the corner of the obstacle car.

In Figure 10, the initial pose was (1.5 m, 1.25 m, 0); the final poses for continuous-curvature path and RL were (−4.60 m, −0.85 m, 4.56°) and (−4.70 m, −0.86 m, 0.73°), respectively. Both the path planning method and the proposed method met the safety requirements. The final angle of RL was notably smaller than that given by the planned path. 

To interpret the differences in the final pose of both methods, the path curvature is compared in Figure 10c. This figure demonstrates that, during the early stage of parking, the proposed reinforcement learning method behaved more decisively than the continuous-curvature method. In the conventional method, the path was designed using a speed of 1 m/s, such that the path could be followed by the path following control modular. If the real speed is below 1 m/s, the path can theoretically be followed [24]. The continuous-curvature method failed to maximize the system performance. In the acceleration phase in Figure 10d, the speed was slower than 1 m/s and the steering wheel action in the time domain resulted in more urgent steering in the distance domain to the first curvature rate change point, as the action of RL is coupled with time. Figure 10c illustrates that the path length of RL was shorter than the planned path using continuous-curvature curves. MCTS searches for the most promising action to maximize the reward Equation (6) at each control interval. The advantage of integrated planning and control in a rolling optimization manner is verified. 

### 5.3. Data Efficiency Verification During Adaptability to Changes in Vehicle Model

We evaluated the sample efficiency of DERL by comparing the parking sample numbers required by DERL and several benchmarks to obtain a stable solution. All the algorithms were based on MCTS and used the same pre-trained policy network. The benchmarks include DERL without adaptive *μ*, data augmentation, and off-policy re-weighting. The evaluation index was the total reward, which is high only if all its component rewards are high. The parameters of the network were updated every 25 trials (one iteration).

The vehicle dynamics model has strong non-linearity at low speed. As in the last section, the trained RL model using the kinematic model was transferred to control the high-fidelity Carsim vehicle. Adaptability to changes in the vehicle model is crucial for automatic parking. To represent a variable vehicle model, the transmission ratio from the steering wheel angle to the front wheel was altered from 15.88 to variable, as shown in Table 2. Similar to the real system, the steering rate was higher at small steering wheel angles.

**Figure 11:**
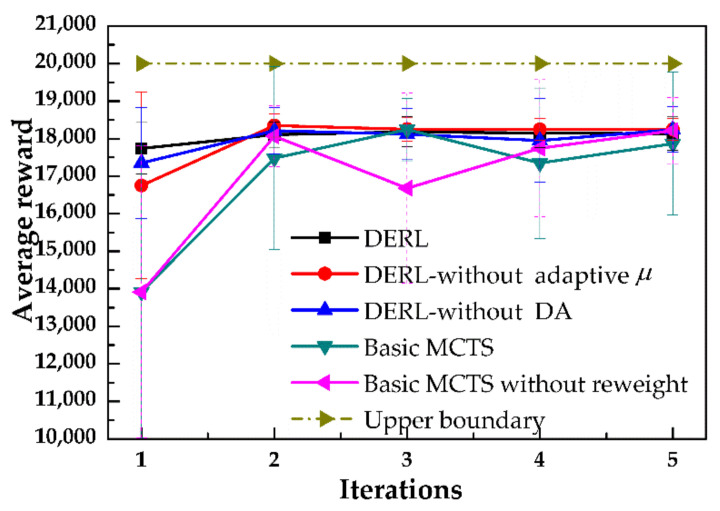
Average reward over 25 initial parking position during the learning processes of different algorithms where DERL is the proposed complete method. One iteration is equal to 25 parking trials.

Figure 11 shows that the proposed method required the least amount of parking samples to achieve the same performance level. The initial policy was closer to the optimal solution; the data augmentation and the guidance of the highly discrete action (Figure 7d) brought benefits to DERL. The basic MCTS without re-weighting did not reuse historical parking experiences in an on-policy manner and the training was unstable for the selected parameter. By contrast, the off-policy re-weighting process smoothed the learning process, as the training data that had high weight among all past experiences were continuously selected from the data set generated by the different policies. For DERL without adaptive *μ* and data augmentation, the former required fewer interactions. This indicated that parking experience augmentation with imagination rollouts had a stronger impact, compared to the guidelines provided by fuzzy action prediction. The standard deviation of the proposed method was lower than those of basic MCTS and MCTS without re-weighting. Both the data augmentation and adaptive policy guidance contributed to the robustness of the method. This confirms that a mismatched vehicle model, within a certain degree, can be carefully used to improve the data efficiency.

**Figure 12:**
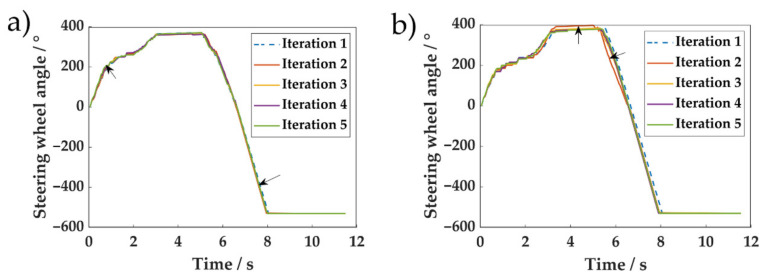
Steering wheel angle for each iteration at initial position (1.25 m, 1.5 m, 0) using different methods: (**a**) Steering angle for the proposed data-efficient reinforcement learning; and (**b**) basic MCTS without re-weighting. The black arrows are the direction of the changes.

To intuitively interpret the results, the steering angle outputs for each iteration are shown in Figure 12. The basic MCTS without the re-weighting process updated the value of the state in an unreliable direction for the second iteration, which led to inconsistent steering wheel action change. In contrast, the proposed method was more stable with each generation. This indicates that the fitting of the value function in the proposed method was more robust to the uncertainty of network training. This phenomenon is similar to that reported by Wang et al. [33], where past experience was used to improve the actor–critic algorithm’s parameter update direction. The result of removing off-policy re-weighting revealed that data from past interactions with the environment are also favorable for AMPI-based reinforcement learning. Compared with the basic MCTS, the reward of DERL with data augmentation rose more quickly. DERL used unbiased estimation of model-generated rollouts as supplementary data, which is different from the mode-free method, in which the performance was sensitive to the model error. In model-free RL, short-range model-generated rollouts branched from the real-world data were demonstrated to avoid the model pitfalls [34]. However, it is not applicable in our case where the rewards are sparse.

Overall, the proposed reinforcement learning method achieved an average reward of 18,106 in 25 trials (i.e., one network update) in Carsim and, so, the data efficiency was verified. Each module of the proposed DERL was supported by the ablation study for its adaptability to changes in the vehicle model.

## 6. Real Vehicle Experiments

**Figure 13:**
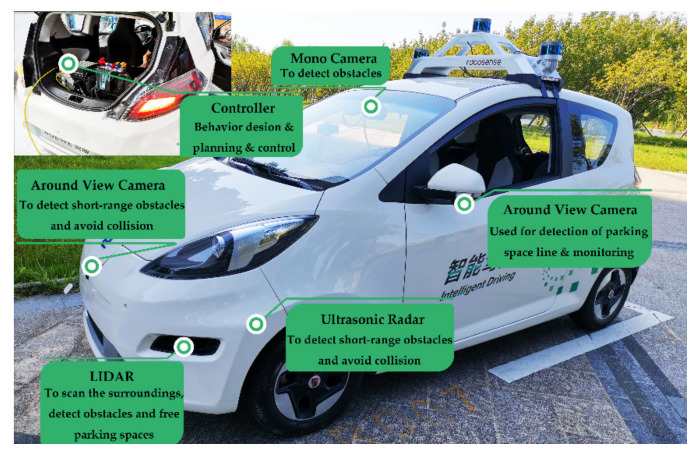
The test platform equipped with AVM, LiDAR and a 900 MHz processor.

A real full-sized vehicle experiment was performed to demonstrate the effectiveness of the proposed method. The initial training positions were the same as in Figure 6. The experimental platform was a Roewe E50 pure electric car made by SAIC Motor (Shanghai, China), as shown in Figure 13. The dimensions of the parking space are 5.5 m × 2 m. The algorithm was executed using a dSPACE MicroAutoBox Ⅱ 1401/1513 with a 900 MHz processor. Steering was controlled by an electronic power steering system. The maximum error of the positioning accuracy during parking was about 0.1 m, using the federated Kalman filter estimation algorithm. As driving torque at low speed struggles to overcome static friction, the starting and termination speed orders were set to step signals of 0.2 and 0 m/s. 

The test platform was equipped with an AVM system, mono camera, LiDAR, radar, and wheel speed sensors. The AVM system monitored around the body, constructing a bird’s-eye view, was used for the detection of parking space lines and remote monitoring [3,4,35]. The mono camera was used to detect pedestrians and other vehicles. The radar was used to detect short-range obstacles. The LiDAR can detect the parking space (i.e., the space between two vehicles) [5]. After the detection of the parking space, the vehicle pose was estimated by dead reckoning, using the data of the motion sensors [19,24]. 

The experiments (also see Supplementary Figure S1, Video S1 and Appendix B) consisted of two groups: First, the initial positions were set as training positions as shown in Figure 6 (25 tests), in order to verify the adaptability of the algorithm to real vehicle dynamics. Second, the initial orientation at (2.5 m, 1.75 m, 0) was changed from 0° to [−10°, 10°] with an interval of 1°, in order to test the generalization ability to unseen initial parking poses for 20 tests. As shown in Table 3, the mean final angle Δ*θ* between the vehicle longitudinal axis and the road edge was less than 1° with a standard deviation of 0.0728°, fulfilling the requirement of ISO 16,787 [36] (−3° to 3°, 1.5°). Compared to parking relying on the pre-trained model (MCTS + policy network) and the pre-trained model with a refined vehicle model, the final pose of the proposed method was better, with respect to a deviation of *y*, final angle, and success rate. Among the three methods, using MCTS plus the policy network and kinematic vehicle model performed the worst, as it suffered from model error. The result shows that a vehicle model with high precision is required to directly apply the order sequence searched by MCTS [10]. Compared with Zhang et al. [10], in which MCTS only used the policy ANN and the refined vehicle model to obtain the reward, DERL does not model the response characteristics of the vehicle steering and driving system, showing less requirement on the accuracy of the vehicle model. However, the potential disadvantages of DERL are its longer run time (about 3 m longer) and more complex structure, which requires more hyper-parameters introduced by the value network.

**Figure 14:**
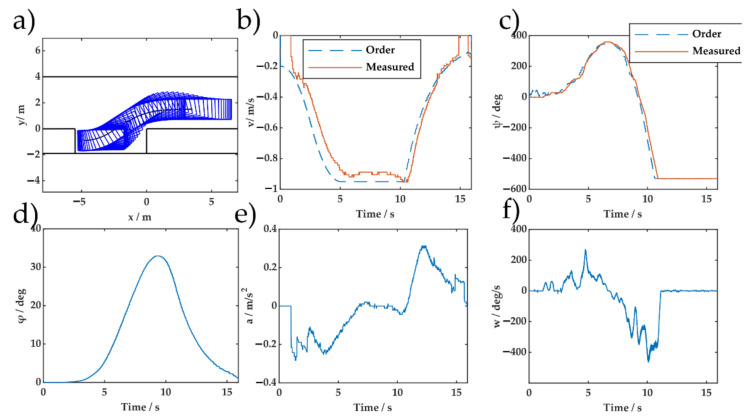
Experimental parking control and state profiles of DERL for the initial pose (3.5 m, 1.5 m, 0): (**a**) Parking trajectory; (**b**) speed profile; (**c**) steering wheel angle profile; (**d**) orientation angle; (**e**) acceleration; and (**f**) steering wheel speed.

To analyze the results, the parking control and state profiles for the initial position (3.5 m, 1.5 m, 0) are shown in Figure 14, which shows that the driving and steering systems had inevitable time delays. Although we did not model this characteristic, the model error does not accumulate for the proposed method, due to its rolling planning and control feature at each control time interval. The adaptability of DERL to vehicle dynamics was confirmed.

The experimental results for different initial poses are shown in Table 4. The results are similar to those in Table 3: the final pose accuracy and stability performance of DERL were better than those of the parking trajectory given by the others. The tree search in DERL acted as a local optimizer. The reactive experience of the policy network and the predictive long-term return of Equation (6) by the value network were combined by MCTS, thus maximizing its overall performance. Similar to the results provided in Table 3, MCTS with the policy network using only the vehicle kinematic model performed the worst, partially due to the deviation in the chassis response (as shown in Figure 14b,c).

**Figure 15:**
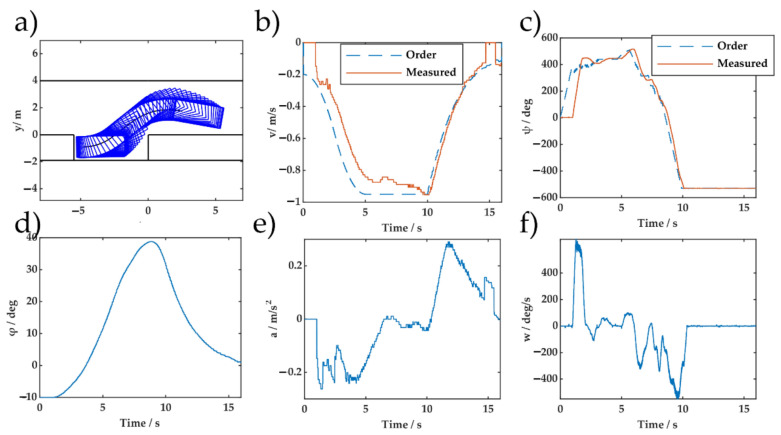
Experimental parking control and state profiles of DERL for the initial pose (2.5 m, 1.75 m, −10°): (**a**) Parking trajectory; (**b**) speed profile; (**c**) steering wheel angle profile; (**d**) orientation angle; (**e**) acceleration; and (**f**) steering wheel speed.

**Figure 16:**
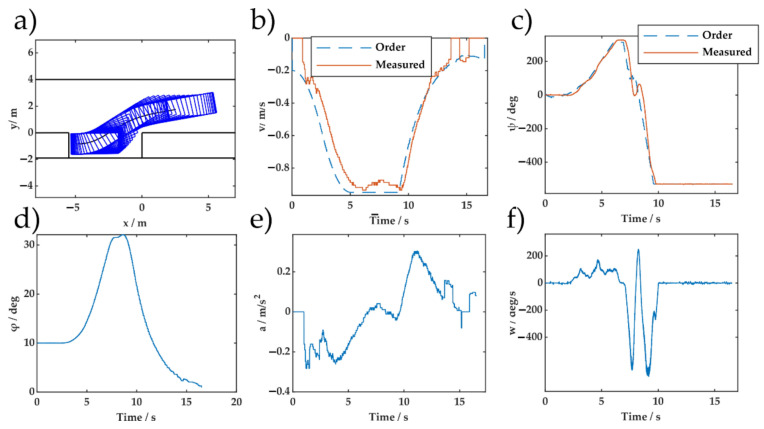
Experimental parking control and state profiles of DERL for the initial pose (2.5 m, 1.75 m, 10°): (**a**) Parking trajectory; (**b**) speed profile; (**c**) steering wheel angle profile; (**d**) orientation angle; (**e**) acceleration; and (**f**) steering wheel speed.

The test control and state profile are shown in Figure 15 and Figure 16, respectively. The generalization ability of the proposed method was confirmed by the experimental results. Both the proposed DERL and the widely used DDPG [8] use off-policy reinforcement learning. The significant difference was that DERL used the vehicle model to extract an unbiased estimate of the state value information using Monte Carlo simulation, which is performed offline. This feature allows DERL to combine real parking information with imaginary rollouts. The integration of the planning algorithm and machine learning is potentially more scalable and effective. Compared with [10], which relied on many data to build and verify the accuracy of vehicle model, DERL uses a kinematic vehicle model without system identification. The advantage of value ANN in MCTS has been confirmed.

## 7. Conclusions

In this work, a novel design for a reinforcement learning algorithm composed of Monte Carlo tree search and two neural networks was proposed for data-efficient automatic parking. First, MCTS guided by an action classification network is used to obtain a pre-trained model. Subsequently, the state value fitting network is trained using the parking data generated by the pre-trained model. Then, the classification network and fitting network are combined with MCTS. If the controlled objective is changed the ANNs can be updated. In the simulation, DERL achieved better parking in a slot within one maneuver, and showed better adaptability than the path-velocity decomposition method. The ability of continuous learning was realized by the use of value ANN. The improvement in data efficiency was confirmed by both high-fidelity Carsim simulations and real vehicle experiments. The key to its success is the integration of Monte Carlo tree search, adaptive action exploration guidance, machine learning, and the use of the vehicle model. DERL demonstrated that model-based reinforcement learning is not only feasible but also scalable and practicable for a safety-crucial autonomous driving task. 

The proposed method generates one control order at each time interval, in a receding horizon manner under time-varying perception errors. That is realized by truncating the simulation in [10]. The location errors are inevitably delivered to the planning and control system, as the latter make decisions based on the perception result. The real vehicle experimental results showed that the final parking performance of DERL, which did not use system identification, was better than that of MCTS guided by a policy network using a refined vehicle model, which planed fewer motions and was more affected by vehicle model errors and perception errors. Similar to [1,8,19], re-planning proved to be a powerful tool for autonomous driving systems under perception uncertainty.

The suggested areas of future studies are threefold: First, the error characteristics of parking space detection and vehicle odometry can be explicitly modeled, and the uncertain information of the motion sensors and the filtering algorithm can be integrated into the tree search, in order to realize probabilistic inferences. The potential method is the likelihood function learning of the multiple-input multiple-output systems [37]. Second, the proposed method can be expanded to maneuver planning, such that the agent can park in smaller spaces; by contrast, the current work only realizes backward motion. Also, the dimensions of the parking space, cross-parking scenarios, as well as irregularly placed obstacles should be further considered. Third, we plan to apply the proposed method to multi-agent interactive parking, where the tracking and behavior prediction of other vehicles are necessary. The proposed model-based data-efficient learning algorithm may serve as an essential component of a larger intelligent system.

## Figures and Tables

**Table 1:** The inputs and outputs of artificial neural networks (ANNs).

Networks	Inputs	Outputs
Value network	Pose (*x*, *y*, *φ*) Steering wheel angle *ψ* Brother number *br*	State value *V_est_*
Policy network	Pose (*x*, *y*, *φ*) Steering wheel angle *ψ*	Angle increment probability distribution ***p***

**Table 2:** Variable steering rate for different steering wheel angle of the controlled vehicle.

Steering Rate										
Steering wheel angle (°)	−530	−430	−330	−230	−130	130	230	330	430	530
Steering rate	15.90	16.11	16.21	16.23	16.53	16.45	16.32	16.20	16.07	15.79

**Table 3:** Test results for the offline training positions of 25 × 3 parking trials.

Variable	DERL		MCTS + Policy ANN + Refined Vehicle Model		MCTS + Policy ANN	
	Mean	Std. Deviation	Mean	Std. Deviation	Mean	Std. Deviation
Δ*y* (m)	0.0224	0.0229	0.0944	0.0267	0.6566	0.3882
Δ*θ* (°)	0.8867	0.0728	2.2475	0.9722	−13.3821	6.4556
Dr (m)	0.0872	0.0297	0.1697	0.0269	−0.5565	0.3711
Df (m)	0.0516	0.0302	0.0793	0.0363	−0.0260	0.1852
Safe d1 (m)	0.2025	0.0314	0.2166	0.0347	0.1124	0.0922
Safe d2 (m)	0.2298	0.0443	0.2643	0.0365	−0.1112	0.1707
Safe d3 (m)	0.2058	0.0434	0.2034	0.0215	0.2457	0.0030
Time (s)	14.9602	0.8968	15.5552	0.7277	14.8658	0.8314
Success (%)	100		84		8	

**Table 4:** Test results for changing initial poses for 20 × 3 parking trials.

Variable	DERL		MCTS + Policy ANN + Refined Vehicle Model		MCTS + Policy ANN	
	Mean	Std. Deviation	Mean	Std. Deviation	Mean	Std. Deviation
Δ*y* (m)	0.0169	0.0148	0.0892	0.0263	0.4389	0.3143
Δ*θ* (°)	0.9038	0.0800	2.8832	2.5573	−7.8702	10.1951
Dr (m)	0.0846	0.0173	0.1603	0.0277	−0.3454	0.3064
Df (m)	0.0485	0.0186	0.0445	0.0854	−0.0311	0.2066
Safe d1 (m)	0.2182	0.0373	0.2178	0.1344	0.1105	0.0902
Safe d2 (m)	0.2162	0.0507	0.2861	0.0719	0.0791	0.4118
Safe d3 (m)	0.1917	0.0502	0.2081	0.0225	0.2906	0.1601
Time (s)	15.0560	0.6546	15.5248	0.3983	14.1632	1.3715
Success (%)	100		70		10	

## Supplementary Materials

The following are available online at https://www.mdpi.com/1424-8220/20/24/7297/s1, Figure S1: All parking trajectory of experiments, Video S1: PARKING20200905.mp4.

## Appendix A

**Table A1:** Whole set of parameters.

**Vehicle Parameters**	
Overall length	3.569 m
Overall width	1.551 m
Front overhang	0.72 m
Rear overhang	0.544 m
Wheel base	2.305 m
Transmission ratio	15.88
**Neural network parameters**	
Size of policy network’s hidden layers	[25, 25]
Size of value network’s hidden layers	[50, 50]
Data divide	{80%, 10%, 10%}
Number of ANNs	10
**Control setup**	
Steering wheel angle resolution	5°
Number of discrete actions	9
Control interval	50 ms
Maximum steering order speed	400°/s
**MCTS**	
*c_puct_*	0–10,000
Sampling times	10–100
*μ*, *τ*	{0.5, 1.5}, 0.1
**Reward function**	
*c*_1_, *c*_2_, *c*_3_	1.1, 0.3, 0.5
**Data augmentation**	
Action repeat numbers	10
Simulation numbers	36

## Appendix B

**Table A2:** Test extreme results for training poses for 25 × 3 parking trials.

Variable	DERL		MCTS + Policy ANN + Refined Vehicle Model		MCTS + Policy ANN	
	Max	Min	Max	Min	Max	Min
Δ*y* (m)	0.0827	0.0021	0.1465	0.037	1.6275	0.0943
Δ*θ* (°)	0.9927	0.7335	5.0014	0.0753	−2.1382	−23.7052
Dr (m)	0.1573	−0.0040	0.2213	0.1115	−0.0192	−1.4875
Df (m)	0.1197	−0.0405	0.1532	−0.0281	0.3293	−0.5608
Safe d1 (m)	0.2975	0.1611	0.3518	0.168	0.4318	0.0154
Safe d2 (m)	0.2986	0.1695	0.3515	0.1993	0.1878	−0.377
Safe d3 (m)	0.2727	0.1453	0.2761	0.1724	0.2492	0.2374
Time (s)	17.875	13.825	16.875	14.285	16.155	13.32

**Table A3:** Test extreme results for changing initial poses for 20 × 3 parking trials.

Variable	DERL		MCTS + Policy ANN + Refined Vehicle Model		MCTS + Policy ANN	
	Max	Min	Max	Min	Max	Min
Δ*y* (m)	0.0474	0.0018	0.1269	0.0374	1.0407	0.0006
Δ*θ* (°)	0.9887	0.6989	8.9211	−0.1239	25.3742	−19.9785
Dr (m)	0.1219	0.0575	0.2046	0.1119	0.1502	−0.9195
Df (m)	0.0874	0.0178	0.1168	−0.1696	0.1541	−0.8375
Safe d1 (m)	0.2930	0.1761	0.5076	0.0104	0.4184	0.0087
Safe d2 (m)	0.2878	0.1203	0.4331	0.1892	1.6144	−0.2839
Safe d3 (m)	0.2610	0.1005	0.2652	0.1783	0.9498	0.238
Time (s)	16.5250	14.175	16.14	14.83	15.085	8.7
