# Supplementary figures and images for: Data Efficient Reinforcement Learning for Integrated Lateral Planning and Control in Automated Parking System
Source: Sensors (Basel). 2020 Dec 18;20(24):7297. doi: 10.3390/s20247297 (PMC7766926; doi:10.3390/s20247297)

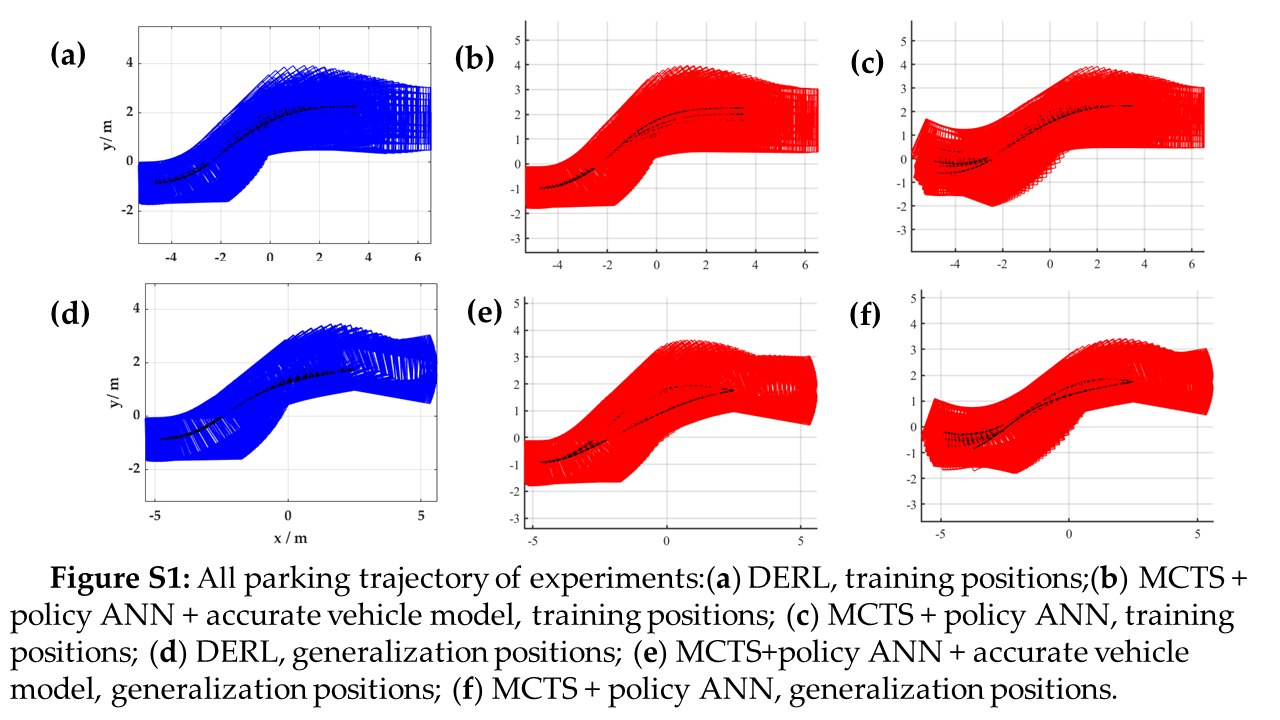

Supplement: Supplementary file 1 [file sensors-20-07297-s001.zip › SupplementaryFile/Figure_S1_All_parking_trajectory_of_experiments.tif]
